# Study on Pear Flowers Detection Performance of YOLO-PEFL Model Trained With Synthetic Target Images

**DOI:** 10.3389/fpls.2022.911473

**Published:** 2022-06-07

**Authors:** Chenglin Wang, Yawei Wang, Suchwen Liu, Guichao Lin, Peng He, Zhaoguo Zhang, Yi Zhou

**Affiliations:** ^1^Faculty of Modern Agricultural Engineering, Kunming University of Science and Technology, Kunming, China; ^2^College of Intelligent Manufacturing Engineering, Chongqing University of Arts and Sciences, Chongqing, China; ^3^School of Mechanical and Electrical Engineering, Zhongkai University of Agriculture and Engineering, Guangzhou, China; ^4^School of Electronic and Information Engineering, Taizhou University, Taizhou, China

**Keywords:** YOLOv4, target detection, pear flowers identification, yield estimation, deep learning

## Abstract

Accurate detection of pear flowers is an important measure for pear orchard yield estimation, which plays a vital role in improving pear yield and predicting pear price trends. This study proposed an improved YOLOv4 model called YOLO-PEFL model for accurate pear flower detection in the natural environment. Pear flower targets were artificially synthesized with pear flower’s surface features. The synthetic pear flower targets and the backgrounds of the original pear flower images were used as the inputs of the YOLO-PEFL model. ShuffleNetv2 embedded by the SENet (Squeeze-and-Excitation Networks) module replacing the original backbone network of the YOLOv4 model formed the backbone of the YOLO-PEFL model. The parameters of the YOLO-PEFL model were fine-tuned to change the size of the initial anchor frame. The experimental results showed that the average precision of the YOLO-PEFL model was 96.71%, the model size was reduced by about 80%, and the average detection speed was 0.027s. Compared with the YOLOv4 model and the YOLOv4-tiny model, the YOLO-PEFL model had better performance in model size, detection accuracy, and detection speed, which effectively reduced the model deployment cost and improved the model efficiency. It implied the proposed YOLO-PEFL model could accurately detect pear flowers with high efficiency in the natural environment.

## Introduction

Yield estimation is an important part of fruit production playing a decisive role in fruit market strategy layout and fruit planting practice (Maldonado and Barbosa, 2016). In the flowering stage of fruit trees, fruit flowers directly reflect the initial number of fruits. The detection of fruit flowers can effectively help orchard owners make management decisions regarding fruit growth to estimate fruit yield and economic benefits (Dias et al., 2018a). Fruit flower identification in the close-up scene is the primary basis for vision-based fruit yield estimation (Wang et al., 2020). Pear with rich nutrition has a huge global output, and accurate identification of pear flowers in the close-up scene is conducive to the prediction of pear output in advance and guides pear fruits to the market.

Fruit flower detection based on vision technology has always been a research hotspot. The algorithm models based on digital image processing contributed to early fruit flower detection (Hoèevar et al., 2014; Aquino et al., 2015; Liu et al., 2018). Most of the early fruit flower detection techniques were used to classify fruit flowers (Lee et al., 2015; Aalaa and Ashraf, 2017). However, ordinary image processing algorithms cannot accurately identify small and dense image targets resulting in low recognition accuracy. Deep learning can extract the essential characteristics of data samples by training a mass of data sets and using a few sample sets to test when identifying sample targets, which has the advantages of strong learning ability and high recognition accuracy. As an emerging field of machine learning research, deep learning has been widely used in agriculture (Yue et al., 2015; Tang et al., 2016; Almogdady et al., 2018). Many fruit flower detections can be performed by using deep learning models including convolutional neural network (CNN), full Convolution Net (FCN), mask region convolutional neural network (Mask R-CNN), etc. (Dias et al., 2018b; Rudolph et al., 2019; Deng Y. et al., 2020). Recently, the application of an improved deep learning model in fruit flower detection has received too much focus to improve the detection accuracy of fruit flowers. A flower detector based on a deep convolution neural network was proposed, which could be used to estimate the flowering intensity and the average accuracy score of the detector was 68% (Farjon et al., 2020). Lin et al. (2020) used the fast regional convolution neural network (Fast R-CNN) model to detect strawberry flowers by combing an improved VGG19 network to represent the multi-scale characteristics of strawberry flowers. Tian et al. (2020) adopted the Mask Scoring R-CNN instance segmentation model with U-Net as the backbone network. According to the unique growth characteristics of apple flowers, ResNet-101 FPN was used to extract the spliced feature map. Their experimental results showed that the accuracy and recall of this method were 96.43 and 95.37%, respectively.

As a representative of one-stage target detection algorithms, you only look once (YOLO) series algorithms are specially characterized by generating candidate regions in the results. Compared with the two-stage target detection algorithm, the biggest advantages of YOLO series algorithms are their very fast running speed and high detection accuracy. The YOLO algorithm has been widely applied, such as defect detection in the industrial field (Deng H. F. et al., 2020; Hui et al., 2021; Li et al., 2021), disease detection in the medical field (Albahli et al., 2020; Abdurahman et al., 2021), detection of railway components and signals in transportation (Guo et al., 2021; Liu et al., 2021), and galaxy detection in the astronomical field (Wang X. Z. et al., 2021). In the agricultural field, YOLO series algorithms are also used to detect diseases and pests (Liu and Wang, 2020). YOLO series of algorithms are currently widely recognized in four versions from YOLOv1 to YOLOv4. Redmon et al. (2016) first proposed a YOLOv1 model that was a single-stage target detection network to realize the requirement of rapid improvement of the detection speed of the target detection algorithm. The YOLOv1 model divides the input image into several grid cells and uses the convolution layer and maximum pooling layer to extract features. The detection speed of the YOLOv1 model is improved, however, the effect of small target detection is not good. Redmon and Farhadi (2017) proposed a YOLOv2 model based on improving the recall rate and positioning accuracy of the YOLOv1 model, which used DarkNet-19 as a feature extraction network with an input layer. Then, the YOLOv2 was improved by changing the backbone network to DarkNet-53, adopting a feature pyramid network in the neck network, and replacing Softmax with logistic regression in the prediction layer. Alexey et al. (2020) proposed a YOLOv4 model to add a variety of techniques to the backbone network and neck network so that the network was more portable and faster to detect. The backbone network of the original YOLOv4 model has too many layers composed of many CSP structures and residual modules. Although the YOLOv4 model conducts accurate detection, the detection running time of the model is far beyond the reach of the real-time detection requirements for light equipment.

The contribution of YOLO algorithms in fruit flower detection focuses on the structural improvement of the YOLO-based deep learning model framework. Wu et al. (2020) used the channel pruning algorithm to reduce the amount of YOLOv4 model parameters and used the manually labeled dataset image to fine-tune the model to realize the real-time and accurate detection of apple flowers in different environments. Their experimental results show that the mAP value of apple blossom detected by the proposed model reached 97.31%. Compared with other five different deep learning algorithms, the mAP value of the proposed model improved by at least 5.67%. For the detection of tea chrysanthemum in a complex natural environment, Qi et al. (2021) designed a lightweight F-YOLO model adopting CSPDesenet as the backbone network and CSPResnet as the neck network. Accurate detection results could be obtained under different conditions by using their proposed F-YOLO model.

Inspired by the above introduction, this study applied the YOLOv4 model to detect pear flowers in the natural environment. ShuffleNetv2 network is a lightweight network with few network layers, which can make greater use of characteristic channels and network capacity in a limited space (Ma et al., 2018). This study proposed a method to replace the backbone network of YOLOv4 to reduce the number of backbone network layers and computational complexity. The pear flower images were synthesized with the visual features of the pear flowers. A new YOLO-PEFL model was constructed by using ShuffleNetv2 embedded by the SENet (Squeeze-and-Excitation Networks) module to replace the original backbone network (CSPDarkNet53) of the YOLOv4 model. The proposed YOLO-PEFL model was trained with synthetic pear flower targets. Experiments were designed to evaluate the performance of the proposed model to achieve the accurate identification of the pear flowers within a short running time.

## Materials and Methods

### Pear Flower Image Acquisition

Pear flower images were acquired from the pear orchard of Yongchuan, Chongqing, China. The longitude and latitude of the pear orchard are 105°52′24′′ east longitude and 29°16′54′′ degrees north latitude, respectively. During the period from March 12, 2020 to March 21, 2020, pear flower pictures were acquired from 10 a.m. to 4 p.m. every day. The pear flower varieties were Huangguan pear flowers and Xiayu pear flowers. The distance between the camera and the pear flowers was 1–2 m. A total of 968 color images of pear flowers were obtained under different lighting conditions by using a Sony digital camera (Tokyo, Japan) and an Apple mobile phone (Cupertino, CA, United States). In total, 467 and 501 color images of pear flowers were acquired by using the Sony digital camera and the Apple mobile phone, respectively. The model of the Sony digital camera is Sony DSC-WX100 and the resolution is 2592×1944. The model of the Apple mobile phone is the iPhone 6s plus and the resolution is 3024×4032.

### YOLO-PEFL Model Construction for Pear Flowers Detection

#### Pear Flower Target Synthesis

**FIGURE 1:**
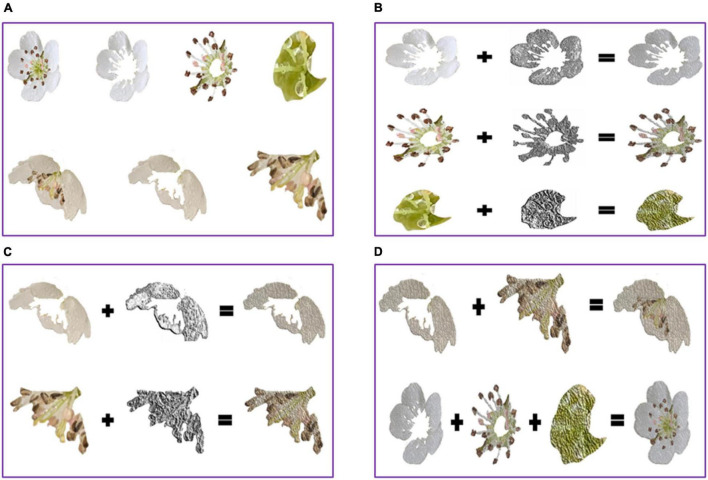
Synthetic pear flower targets. **(A)** Two forms of pear flower targets with the divided pear flower parts; **(B)** the recombined parts of the pear flower target with the flower core; **(C)** the recombined parts of the pear flower target without the flower core; **(D)** two synthetic pear flower targets.

The pear flower targets were artificially extracted from the pear flower images using photoshop. They presented two forms visually. One form was that the flower core could be seen in the first picture of the first row of Figure 1A. The other form did not present the flower core shown in the first picture of the second row of Figure 1A. The pear flower target with the flower core was artificially divided into the petal, the anther, and the flower core shown in the second picture, the third picture, and the fourth picture of the first row of Figure 1A, respectively. The pear flower target without the flower core was artificially divided into the petal and the anther shown in the second picture and the third picture of the second row of Figure 1A, respectively. The local binary pattern (LBP) operator was used to extract the texture features of the pear flower targets. Petal, anther, and flower core images were recombined by overlapping their respective LBP texture feature images on their original images. As shown in Figures 1B,C, the third column presented the recombined petal, anther, and flower core of the pear flower target, the first column included the original images of the petal, anther, and flower core, and the second column presented the LBP texture feature images of the petal, anther, and flower core. Finally, two forms of pear flower targets were re-synthesized by combining the recombined petal, anther, and flower core shown in the third column of Figure 1D.

#### Composition of Pear Flower Image Data

**FIGURE 2:**
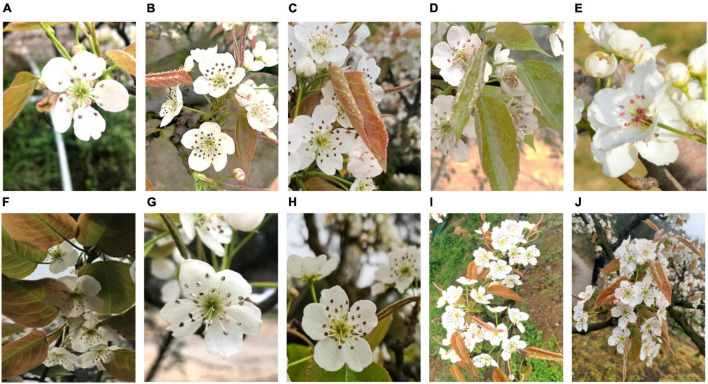
Natural images of pear flowers. **(A)** Single flower; **(B)** multiple flowers; **(C)** slight occlusion; **(D)** serious occlusion; **(E)** front illumination; **(F)** back illumination; **(G)** sunny day; **(H)** cloudy day; **(I)** multiple flowers with serious occlusion in sunny day; **(J)** multiple flowers with serious occlusion in cloudy day.

**FIGURE 3:**
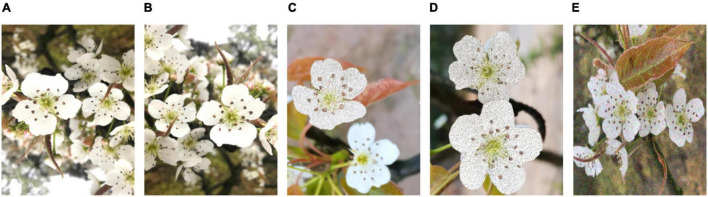
Augmentation images of pear flowers. **(A)** Inversion image; **(B)** mirror image; **(C)** image including partially synthesized pear flowers; **(D)** image including all synthesized pear flowers; **(E)** image including noises.

The dataset of pear flower images used in this study consists of pear flower images in the natural environment and artificially augmented pear flower images shown in Figures 2, 3, respectively. The natural images of pear flowers mainly include the images of single pear flowers, the images of multiple pear flowers, the images of pear flowers being slightly occluded, the images of pear flowers being seriously occluded, the images of front illumination, the images of back illumination, the images of pear flowers in sunny day, the images of pear flowers in a cloudy day, the images of pear flowers being seriously occluded in sunny day, and the images of pear flowers being seriously occluded in a cloudy day. Their representative images are shown in order from Figures 2A–J. The artificially augmented pear flower pictures consist of the inversion images of pear flowers, the mirror images of pear flowers, the images including partially synthesized pear flowers, the images including all synthesized pear flowers, and the images including noises, the representative one of which are presented in the order in Figures 3A–E. The corresponding number of pear flower images is recorded in Table 1. The pear flower images were processed into Pascal VOC format and the targets of pear flowers were labeled using an opening source tool “labelimg.” The dataset was randomly divided into a training set, a validation set, and a test set according to the proportions 70, 15, and 15%, respectively.

**TABLE 1:** Information on pear flowers image dataset.

Pear flowers images	Image type	467 images obtained by a Sony digital camera (2592×1944)	501 images obtained by an Apple mobile phone (3024×4032)
Natural image	Single flower	34	30
	Multiple flowers	30	32
	Slight occlusion	29	34
	Serious occlusion	30	39
	Front illumination	34	30
	Back illumination	38	35
	Sunny day	27	33
	Cloudy day	39	33
	Multiple flowers with serious occlusion on a sunny day	42	39
	Multiple flowers with serious occlusion on a cloudy day	40	42
Augmentation image	Inversion image	22	30
	Mirror image	24	31
	Image including partially synthesized pear flowers	28	29
	Image including all synthesized pear flowers	25	30
	Image including noises	25	34

#### YOLOv4 Model-Based Pear Flowers Detection

**FIGURE 4:**
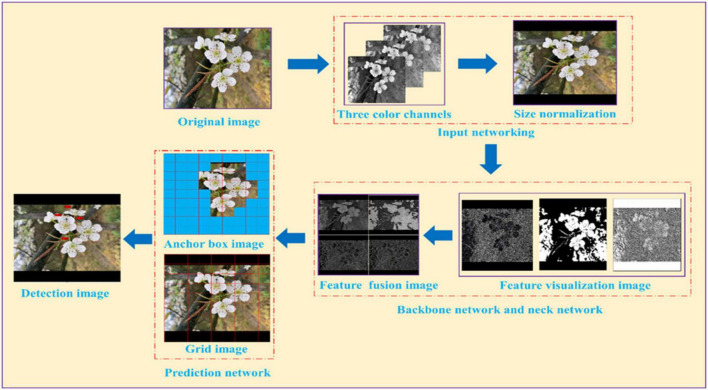
The training process of the YOLOv4 model for pear flower detection.

In order to reduce the computation amount of YOLO series algorithms and ensure algorithm accuracy, Bochkovskiy et al. (2020) proposed the YOLOv4 model by adding various technologies to the overall structure of the YOLOv3 model. The YOLOv4 model includes four main structures of the input network, backbone network, neck network, and head network. The training process of the YOLOv4 model for pear flower detection can be seen in Figure 4. An original image of a pear flower is divided into three color channels and then normalized to a size of 416*416 after entering into the input network. After data enhancement processing, the size normalization image is input to the backbone network and the neck network, where feature images can be obtained by feature visualization, and then the feature images are fused by a series of operations to enhance pear flowers’ features. In the prediction network, the original image of the pear flower is evenly divided by 9*9 grids. Because the size of pear flowers in each image is different, the YOLOv4 model assigns three scale anchor frames to each image to detect pear flowers. Each scale anchor frame has three different sizes. When the center of the pear flower falls into the anchor frames, the anchor frames will lock the target. In the detection image, the boundary frames with confidence can be obtained by optimizing anchor frames of different sizes on the surface of pear flowers.

#### YOLO-PEFL Model-Based Pear Flowers Detection

**FIGURE 5:**
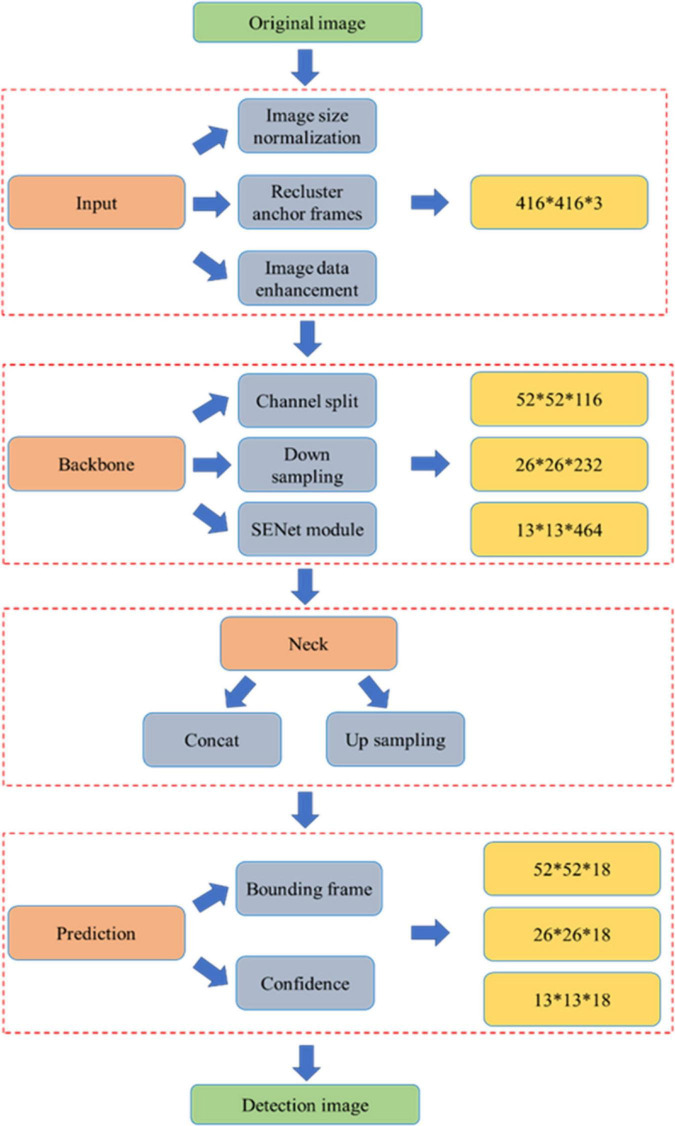
The structure diagram of the YOLO-PEFL model.

The overall structure of the proposed YOLO-PEFL model is shown in Figure 5, which replaces the backbone network of the YOLOv4 model with the ShuffleNetv2 combined with the SENet model including channel split and down sampling. After the original image is preprocessed in the input network, the channel separation operation is implemented on it in the backbone network. When the stride is 1, the channel number of the image remains unchanged. When the stride is 2, the backbone network performs down sampling, and the channel number of the image will be halved. The feature extraction of the image can be improved by embedding the SENet module. The operations of concatenating and up sampling are conducted on the image to fuse corresponding features in the neck network. Thus, the detection image including boundary frames and confidence can be obtained after outputting from the prediction network.

**FIGURE 6:**
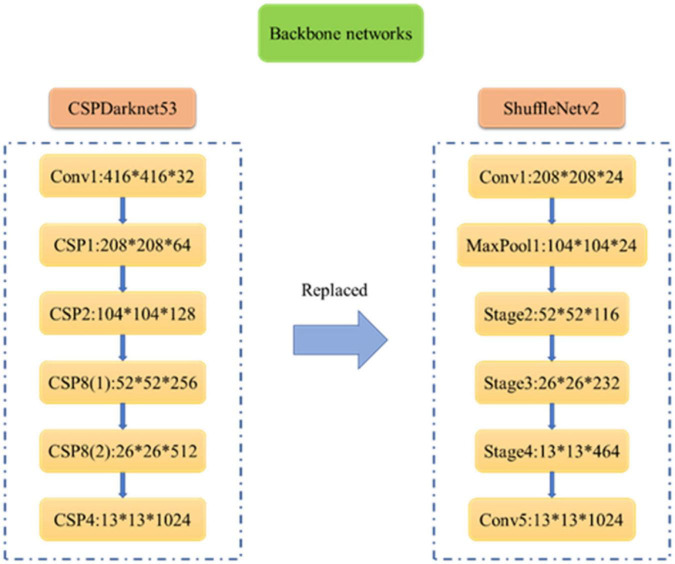
Backbone networks of YOLOv4 model and YOLO-PEFL model.

The specific structure of the backbone network of the YOLO-PEFL model is shown in Figure 6. CSPDarknet53 is the backbone network of the YOLOv4 model, which includes multiple CSP modules to conduct complex group convolution operations. However, the backbone network Shufflenetv2 of the YOLO-PEFL model mainly includes stage modules for point convolution operations, which greatly reduces the amount of module computation.

**FIGURE 7:**
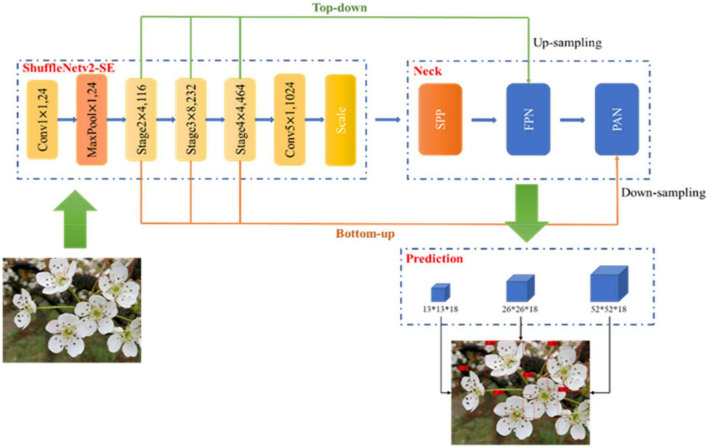
The training process of the YOLO-PEFL model for pear flower detection.

As shown in Figure 7, the overall training process of the YOLO-PEFL network is the same as that of the YOLOv4 network. After size normalization and channel separation processing in the input network, the pear flower image is input into the backbone network with ShuffleNetv2 as the main body. In the backbone network, the pear flower image is first processed by convolution and maximum pooling with a stride size of 2. The output channel of the pear flower image becomes 24, and the image size is reduced by half. Then, the ShuffleNetv2 network obtains image features from three different stage layers by using top-down and bottom-up methods. After processing by the different stage layers, the number of output channels of the image changes to 1,024 combined with the processing of the convolution layer with a stride size of 1. The SENet module adding to the end of the Shufflenetv2 network conducts scale weighting and establishment of nonlinear channel relations. The neck network uses two structures of FPN and PAN to fuse the image features obtained by up-sampling and down-sampling. The detection image including the boundary frames with confidence can be obtained by using three scale anchor frames to detect pear flower targets in the prediction layer.

## Results and Discussion

Two experiments were conducted to verify the performance of the proposed mothed. One was to test the performance of the proposed YOLO-PEFL model after training with the data set containing synthetic pear flower targets and the data set only containing natural pear flower targets, respectively. The other experiment was the comparison of pear flower detection performance of the YOLO-PEFL model, the YOLOv4 model, and the YOLO-tiny model after training with the data set containing synthetic pear flower targets.

The experimental simulation hardware was mainly a laptop computer equipped with an Intel i7-9750h processor, an 16G RAM, and a GeForce GTX 1660 Ti chip. The laptop used the CUDA 10.2 parallel computing architecture and the NVIDIA cudnn7.6.5 GPU acceleration library. The simulation environment was run under the software system of the Darknet/PyTorch deep learning framework (Python version 3.8). MATLAB R2020b, Ashampoo Photo Commander, and Photoshop were used to preprocess the image data. Anaconda, PyCharm, and Visual Studio 2019 were applied to compile and run programs.

### Model Performance Metrics

Model performance metrics reflect the model performance, which mainly includes P (precision), R (recall), F1 (harmonic average), and AP (average precision) shown as Eqs. 1, 3.


(1) $$\left\{ \begin{array}{c} p\,r\,e\,c\,i\,s\,i\,o\,n=\frac{T_{p}}{T_{p}+F_{p}}\\ r\,e\,c\,a\,l\,l=\frac{T_{p}}{T_{p}+F_{N}}\\ F_{1}=\frac{2\times p\,r\,e\,c\,i\,s\,i\,o\,n\times r\,e\,c\,a\,l\,l}{p\,r\,e\,c\,i\,s\,i\,o\,n+r\,e\,c\,a\,l\,l} \end{array}\right.$$

(2) $$A\,P=\frac{\sum p\,r\,e\,c\,i\,s\,i\,o\,n}{N}$$

(3) $$m\,A\,P=\frac{\sum A\,P}{N_{\text{C}}}$$

where *T*_*p*_ represents the number of pear flowers correctly detected, *F*_*p*_ is the number of non-pear flowers incorrectly detected as pear flowers, *F*_*N*_ represents the number of pear flowers that have been missed, N represents the total number of images, and *N*_*C*_ is the number of categories of detected targets. AP representing the integral of accuracy rate to recall rate is equal to the area under the P-R curve directly reflecting the model detection accuracy. mAP is the average of the average precision of all categories in the dataset. Since only one category of target needs to be detected, mAP is equal to AP in this study.

### Detection Performance of YOLO-PEFL Based on Different Data

**FIGURE 8:**
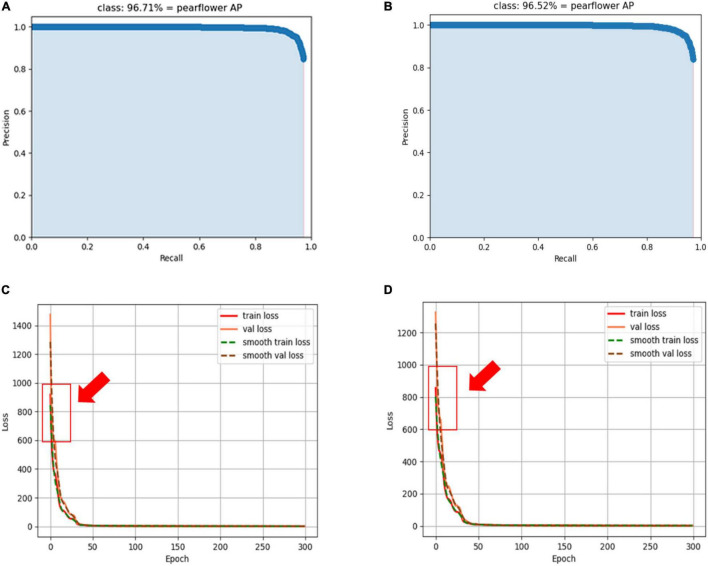
AP curves and loss convergence curves of the YOLO-PEFL model. **(A,C)** Curves obtained after training with synthetic pear flowers targets images; **(B,D)** curves obtained after training with only natural pear flowers targets images.

**FIGURE 9:**
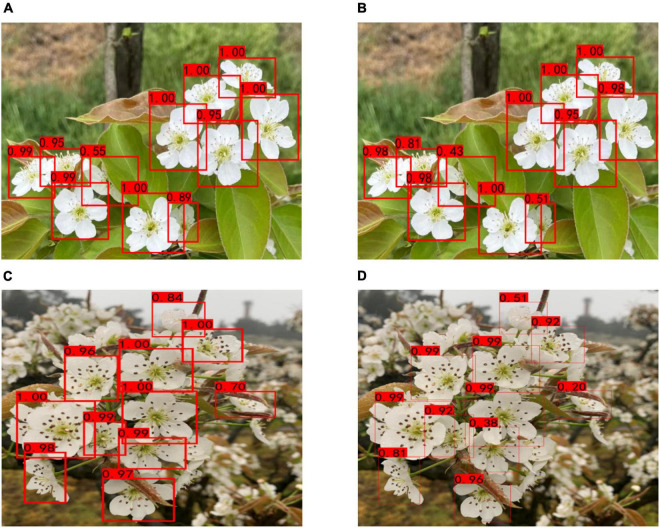
Pear flowers detection results of YOLO-PEFL model after training with different data sets. **(A,C)** Detection results after training with the data set containing synthetic pear flower targets under cloudy and sunny days; **(B,D)** detection results after training with the data set only containing natural pear flower targets under cloudy and sunny days.

After training with the data set containing synthetic pear flowers targets and the data set only containing natural pear flowers targets, respectively, the AP curves and loss convergence curves, pear flowers detection results, and model performance metrics of the proposed YOLO-PEFL model are shown in Figures 8, 9 and Table 2, respectively.

**TABLE 2:** Model performance metrics of three models trained with different data.

Models	Training data sets	P/%	R/%	F1/%	AP/%
YOLO-PEFL	Containing synthetic targets	96.44	92.86	95.00	96.71
	Only containing natural targets	96.34	92.45	94.00	96.52
YOLOv4	Containing synthetic targets	90.84	92.68	92.00	94.95
	Only containing natural targets	90.34	92.23	91.28	94.25
YOLOv4-tiny	Containing synthetic targets	91.97	90.71	91.00	89.80
	Only containing natural targets	91.34	89.97	90.64	89.72

By comparing Figures 8A,B, it was shown that the AP value of 96.71% obtained by using the data containing the synthetic pear flowers targets to train the YOLO-PEFL model was higher than 96.52% obtained by using the data only containing the natural pear flowers targets to train the YOLO-PEFL model. It shows that the detection accuracy of pear flowers could be improved by using the YOLO-PEFL model trained with the data set containing synthetic pear flower targets. After training with two different data, two groups of the loss convergence curves of the YOLO-PEFL model were shown in Figures 8C,D, where the train loss curve and val loss curve represented the change values of the loss function in the training and testing, respectively, and smooth loss curves were the operations of smoothing the curves (Yu et al., 2021). It can be seen in Figure 8 that the loss curves of YOLO-PEFL model training with two data sets decreased rapidly, the curves converged stably to 0, and the coincidence degree of the four loss curves of each group was high. These results implied that the structure of the YOLO-PEFL model using the proposed network to replace the backbone network of the YOLOv4 model was stable. Similar results on the relationship between the stability of the improved model and the variation trend of the loss curves were reported by Suo et al. (2021). However, the only difference between the two groups of loss curves was that the coincidence degree of the four loss curves obtained after training with natural images was not as good as that obtained after training with synthetic images in the red box in Figure 8, which confirmed the YOLO-PEFL model conducted the training and testing well after training with the data containing the synthetic pear flowers targets again.

As can be seen from Figure 9, pear flowers detection had high confidence using the YOLO-PEFL model trained with the two different data sets under both sunny and cloudy days. However, the pear flowers detection confidence of the YOLO-PEFL model trained with synthetic pear flowers data set was higher than that obtained by training with natural pear flowers target data set in the case of severe occlusion. For example, the confidence of an occluded pear flower in Figure 9A was 0.89, which was higher than the confidence of 0.51 in Figure 9B. A similar situation occurred in Figures 9C,D, the confidence of a pear flower seriously obscured by leaves was 0.70 as shown in Figure 9C, which was higher than the confidence of 0.20 of the flower shown in Figure 9D. The above results implied that the YOLO-PEFL model was effective for pear flower detection and the pear flower detection confidence could be improved after training with the data set containing synthetic pear flower targets.

It could be seen from Table 2 that the performance metrics of three models obtained after training with the data set containing synthetic targets were all higher than those obtained after training with the data set only containing natural targets. Among the three models, the YOLO-PEFL model had the highest detection performance metrics after training based on two different data sets. By training with the data set containing synthetic targets, the precision rate, the recall rate, the F1 rate, and the AP of the YOLO-PEFL model were 0.10% higher, 0.41% higher, 1% higher, and 0.21% higher than those obtained after training with the data set only containing natural targets, respectively. They implied the YOLO-PEFL model had the best detection performance of the pear flowers and the detection performance can be improved by training with the data set containing synthetic pear flower targets.

### Convergence Performance Comparison of Three Models

**FIGURE 10:**
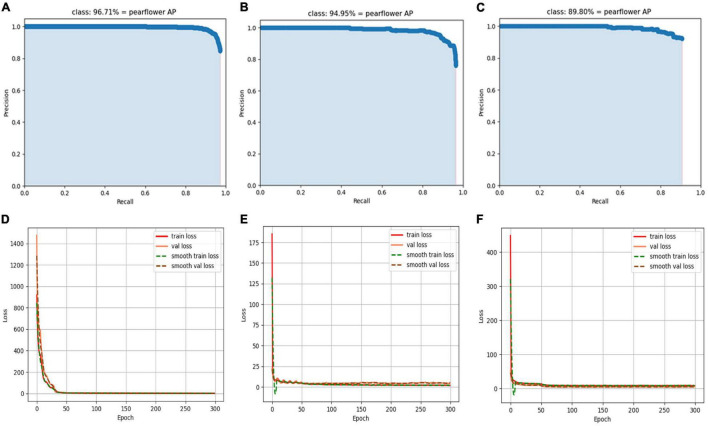
AP curves and loss convergence curves of different models. **(A,D)** AP curve and loss convergence curve of YOLO-PEFL model; **(B,E)** AP curve and loss convergence curve of YOLOv4 model; **(C,F)** AP curve and loss convergence curve of YOLOv4-tiny model.

The training parameters of the three models in the experiment were shown in Table 3. AP curves and loss convergence curves of the three models could be obtained after training with the data set containing synthetic pear flower targets shown in Figure 10.

**TABLE 3:** Parameters setting of three models.

Parameter configuration	YOLOv4	YOLOv4-tiny	YOLO-PEFL
Pre-train models	True	True	False
Random	False	False	False
Initialize learning rate	0.0013	0.005	0.005
Momentum	0.949	0.92	0.92
Decay	0.0005	0.0005	0.0005
Batch size	5	5	8
Epoch	300	300	300

In the test stage, the cut-off values of the three models need to be appropriately selected with the aim of values recall and precision in achieving the best values. In this study, the cut-off value of the three models is selected as 0.5, so that the AP value of the three models reaches the maximum value of their respective models. As shown in Figures 10A–C, the recall upper limit values of the three models are different, which implies detection performance differences of the three models based on different model structures. By comparing Figures 10A–C, it could be found that the YOLO-PEFL model had the highest AP value, which was 1.76% higher than that of the YOLOv4 model, 6.91% higher than that of YOLOv4-tiny model, respectively. It indicated that the YOLO-PEFL model had the highest accuracy of pear flower detection. As shown in Figures 10D–F, the proposed YOLO-PEFL model did not converge until 30 iterations and the convergence speed was slow compared with the other two models. However, the YOLO-PEFL model had the lowest final convergence value which was lower than those obtained by the other two models. The fast convergence speed meant that the model was easy to train, and the low convergence value indicated that the model had good performance (Shi et al., 2021). The smooth training loss curves of the YOLOv4 model and YOLOv4-tiny model fluctuated obviously, which implied that the difference between the predicted value and the ground truth varies greatly (Yan et al., 2021). Thus, the comparison results of Figures 10D–F showed that the YOLO-PEFL model had the best detection performance. These results showed that the backbone network of the proposed YOLO-PEFL model was well-connected with the other three networks of the YOLOv4 model. The overall performance of the YOLO-PEFL model operation was improved compared with the YOLOv4 model.

### Comparison of Detection Results of Three Models

On cloudy and sunny days, the detection results of multi pear flower targets with severe occlusion were as follows using the three models.

**FIGURE 11:**
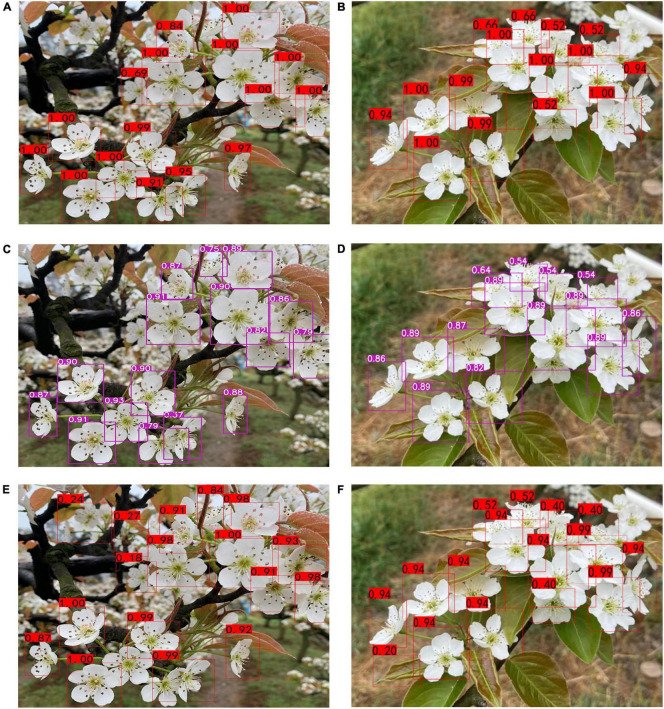
Pear flower detection results based on different models. **(A,B)** Detection results based on the YOLO-PEFL model under cloudy days and sunny days; **(C,D)** detection results based on the YOLO-tiny model under cloudy days and sunny days; **(E,F)** detection results based on YOLOv4 model under cloudy days and sunny days.

As can be seen from Figure 11, there was no significant difference in the detection confidence of pear flowers between sunny and cloudy days, which reflected the high detection stability of the model. The YOLO-PEFL model had higher confidence in the detection of pear flowers for the same pear flower images compared with the other two models. By using the YOLO-PEFL model, the detection confidence of some pear flowers could be as high as 1.00. Due to its simple network construction, the YOLOv4-tiny model had the lowest confidence in pear flower detection, especially in the mutual occlusion of pear flowers. Wang L. et al. (2021) also reported the disadvantage of the low detection rate of the YOLOv4-tiny model in complex environments with mutual occlusion in their blueberry recognition research. The detection effect of the YOLOv4 model was better than that of the YOLOV4-tiny model. Similar results have been confirmed in the report of Fan et al. (2022). However, although the YOLOv4 model had the most complex network structure, its confidence in pear flower detection was not higher than that obtained by the YOLO-PEFL model. When multiple pear flowers blocked each other, its detection confidence was lower than that of the YOLO-PEFL model. Some false detections occurred in the detection based on the YOLOv4 model, such as mistaking leaves for pear flowers and overlapping pear flowers as the same one. These conclusions also could be found in the studies by Lawal (2021); Gai et al. (2021), and Wang et al. (2022). By simplifying the network architecture and improving the backbone network of the YOLOv4 model, the proposed YOLO-PEFL model had the best detection effect in effectively solving the problem of missing detection and error detection.

### Model Performance Metrics Comparison

In Table 4, the precision rate of the YOLO-PEFL model was 96.44%, which was significantly higher than 90.84% of the YOLOv4 model and 91.97% of the YOLOv4-tiny model. The recall rate of the YOLO-PEFL model was 92.86%, which was higher than 92.68% of the YOLOv4 model and 2.15% higher than 90.71% of the YOLOv4-tiny model. Due to the increase in precision and recall rate, the F1 rate of the YOLO-PEFL model reached 95.00% by calculating from Eq. 1, which was bigger than 92.00% of the YOLOv4 model and 91.00% of the YOLOv4-tiny model. AP rates of the YOLOv4, YOLOv4-tiny, and YOLO-PEFL models were 94.95, 89.80, and 96.71%, respectively. The statistical results showed that the proposed model had the highest accuracy in pear flower detection compared with the other two models. The sizes of the three models were 245MB, 22.4 MB, and 42.4 MB, respectively. The size of the YOLO-PEFL model was 82.69% smaller than that of YOLOv4, and 47% larger than that of YOLOv4-tiny. The number of parameters of the model was proportional to the size of the model. The number of parameters of the YOLO-PEFL model was larger than that of the YOLOV4-tiny model but smaller than that of the YOLOv4 model. The YOLOv4 model had the longest training speed of 6.68 h, which was much slower than 2.67 h of the YOLO-PEFL model and 1.53 h of the YOLOv4-tiny model. By comparing the average image detection speed, the detection speed of the YOLO-PEFL model was 0.027s, which was 0.01s faster than that of the YOLOV4 model and 0.02s slower than that of the YOLOv4-tiny model. The above results showed that the proposed model was smaller in size, faster in detection speed, and could achieve a high detection accuracy. The detection speed of the proposed model was suitable for the real-time detection requirements of a general GPU graphics card, which could provide theoretical support for the yield prediction of a pear orchard.

**TABLE 4:** Comparison of evaluation indexes of different models.

Model	P/%	R/%	F1/%	AP/%	Total params	Model size/MB	Training speed/h	Detection speed/s
YOLOv4	90.84	92.68	92.00	94.95	64,040,001	245	6.68	0.036
YOLOv4-tiny	91.97	90.71	91.00	89.80	5,961,014	22.4	1.53	0.008
YOLO-PEFL	96.44	92.86	95.00	96.71	9,885,129	42.4	2.67	0.027

## Conclusion

In this study, an accurate pear flower detection method was proposed, which used the YOLO-PEFL model trained with the data set containing synthetic pear flower targets to detect pear flowers in the natural environment. The main conclusions could be obtained as follows:

(1) A YOLO-PEFL model was constructed using the ShuffleNetv2 embedded by the SENet module to replace the backbone network of the YOLOv4 model.
(2) The performance metrics of pear flower detection of the YOLO-PEFL model could be comprehensively improved by training with the data set containing synthetic targets.
(3) The YOLO-PEFL model had greatly improved the pear flowers detection performance of the YOLOv4 model.
(4) By training with the data set containing synthetic pear flowers targets, the YOLO-PEFL model had a precision rate of 96.44%, a recall rate of 92.86%, an F1 rate of 95.00%, an average precision rate of 96.71%, and an average detection speed of 0.027s, which concludes that the proposed method can accurately detect pear flowers in the natural environment.

Our proposed method using targets of features synthesis to train the deep learning network may also be applicable to the detection of other fruit flowers, and research will be conducted to identify dense small flowers in the future.

## Data Availability Statement

The original contributions presented in the study are included in the article/supplementary material, further inquiries can be directed to the corresponding authors.

## Author Contributions

CW: data curation, investigation, and writing-original draft. YW and SL: writing-review and editing. GL: conceptualization, data curation, methodology, and supervision. PH: investigation, methodology, and writing-review and editing. ZZ: methodology and supervision. YZ: experiment and data curation. All authors contributed to the article and approved the submitted version.

## Conflict of Interest

The authors declare that the research was conducted in the absence of any commercial or financial relationships that could be construed as a potential conflict of interest.

## Publisher’s Note

All claims expressed in this article are solely those of the authors and do not necessarily represent those of their affiliated organizations, or those of the publisher, the editors and the reviewers. Any product that may be evaluated in this article, or claim that may be made by its manufacturer, is not guaranteed or endorsed by the publisher.
